# Addressing the knowledge gap: development of stakeholder-informed training to improve the inclusion of adults with impaired capacity to consent in trials

**DOI:** 10.1186/s13063-025-09182-1

**Published:** 2025-10-22

**Authors:** Victoria Shepherd, Martina Svobodova, Nicola Ivins, Amy M. Russell, Anna Volkmer, Mark Jayes, Kerry Woolfall, Madeleine Clout, Kim Munnery, Shaun Treweek

**Affiliations:** 1 Centre for Trials Research, Cardiff University, 6 Floor Neuadd Meirionnydd, Heath Park, Cardiff, CF14 4YS UK; 2 Leeds Institute of Health Sciences, University of Leeds, Leeds, UK; 3 Division of Psychology and Language Sciences, University College London, London, UK; 4 Faculty of Health and Education, Manchester Metropolitan University, Manchester, UK; 5 Institute of Population Health, University of Liverpool, Liverpool, UK; 6 Bristol Medical School, Bristol Trials Centre, University of Bristol, Bristol, UK; 7 Aberdeen Centre for Evaluation, University of Aberdeen, Aberdeen, UK

**Keywords:** Clinical trials, Inclusivity, Under-served groups, Adults lacking capacity to consent, Cognitive impairment

## Abstract

**Background:**

Improving the inclusion of under-served groups in clinical trials is increasingly being seen as a priority area for research funders and regulators. Adults who lack capacity to make an informed decision about taking part in trials are recognised as an under-served group. Researchers struggle to navigate the complex ethical, legal, and methodological issues surrounding trials involving adults lacking capacity to consent, leading to frequent exclusion of this population. Researchers have identified a need for greater knowledge about designing and conducting trials involving this population. Building on the CONSULT research programme, we developed stakeholder-informed training to help researchers design more inclusive trials.

**Methods:**

The CONSULT e-learning was developed in collaboration with a group of researchers with topic expertise and a lay advisory group with lived experience. It was developed over four phases: (1) establishing researchers’ training needs using an online survey; (2) developing the e-learning content including illustrative case studies, videos, and links to resources and further reading; (3) iterative piloting and refining of the content; (4) dissemination of the e-learning and initial evaluation. A set of informational materials about the e-learning were also developed.

**Results:**

Informed by the stakeholder survey (*n* = 82), the CONSULT e-learning consists of four key modules covering the legal and ethical frameworks, consent and consultation processes, and methodological considerations, with the key role of public involvement threaded throughout. It was launched at a webinar (December 2024), with a post-webinar survey (*n* = 29) showing an increase in awareness about the importance of including adults lacking capacity in trials where they are a relevant population. Researchers also signalled their commitment to changing their research practice, suggesting that the e-learning has a role in facilitating greater inclusion of this under-served population in trials. The CONSULT e-learning is available online: www.capacityconsentresearch.com/training.

**Conclusions:**

Alongside tools such as the INCLUDE Impaired Capacity to Consent Framework, the CONSULT e-learning course aims to support researchers to develop the knowledge and skills needed to design and conduct higher-quality trials that are more inclusive of adults who lack capacity to consent. Further engagement, including with funders who increasingly require inclusion as a condition of funding, is needed.

**Supplementary Information:**

The online version contains supplementary material available at 10.1186/s13063-025-09182-1.

## Background

Trials involving adults with impaired capacity to consent are known to be challenging, which can result in exclusion of this population [1, 2]. This exclusion is a global issue and is widespread, spanning populations such as people living with dementia [3], severe mental health conditions [4], stroke [5], learning disability [6, 7], and those at the end of life [8]. The resulting lack of evidence underpinning the care these populations receive has been recognised in the new WHO guidance on clinical trials [9] and by the Nuffield Council on Bioethics [10]. Improving the inclusion of under-served groups is an international priority for research funders, such as the UK’s National Institute for Health and Care Research (NIHR) which now has research inclusion as a condition of funding [11], and has led to a raft of initiatives to address this issue, including the NIHR’s INCLUDE project [12]. There are also a range of educational and other resources to support the research community to make changes to their research practice as required, such as Trial Forge (https://www.trialforge.org) and STEP-UP (https://step-up-clinical-trials.co.uk) with comparable international resources, to help address the general, context-specific, and intersectional barriers to inclusion.

Whilst there are several challenges associated with conducting research with adults with impaired capacity to consent [13, 14], researchers report that the root of this exclusion is a lack of knowledge about the additional legal, ethical, and methodological issues in trials involving this population [15, 16]. This has led to calls for better support for researchers and others involved in designing, approving, and conducting research which involves (or should involve) people with cognitive disabilities including those with impaired capacity to consent [8, 15, 17].

As part of a larger programme of research exploring the ethical and methodological challenges of conducting research with adults who lack capacity to consent (CONSULT) [18], several tools were developed, such as the NIHR INCLUDE Impaired Capacity to Consent Framework to help researchers to design more inclusive studies [19]. During the implementation period of the framework, researchers identified a need for better training on trials to include people with impaired capacity, to help equip them to use these tools [19].

This paper reports the development of an e-learning resource for researchers and other groups on the design and conduct of trials involving adults with impaired capacity, with the aim of ensuring that this under-served population has greater opportunities to participate in, and benefit from, research. The CONSULT e-learning is available online at www.capacityconsentresearch.com/training.

## Methods

Building on the CONSULT research programme, this was a mixed-methods project designed to understand researchers’ training needs and develop a stakeholder-informed e-learning package to address them. The objectives of the project were to:

1. Identify the training, education, and support needs of researchers who design and conduct trials involving adults with impaired capacity to consent
2. Develop stakeholder-informed e-learning to support researchers to design and conduct trials that are more inclusive of adults with impaired capacity to consent
3. Pilot and implement the e-learning resource, and evaluate its impact

It was conducted in conjunction with a lay advisory group of seven people with experience of living with and/or caring for someone with a condition which can affect decision-making capacity (e.g. dementia), and a researcher advisory group of six researchers with wide-ranging subject matter expertise (e.g. trials methodology, trial management, speech and language therapy, emergency research), and research involving populations who may experience impaired capacity to consent (e.g. people living with dementia, people with learning disabilities, and people with communication disabilities). It was underpinned by adult learning theories such as self-directed learning [20], and informed by a ‘3i’ stakeholder analysis to identify relevant parties who would have an interest in, influence over, and likely be impacted by the project [21]. For the initial phases of the project, the main stakeholders were considered to be researchers and research teams who conduct research involving populations who do/may experience impaired capacity as they have the highest interest in the training and are most likely to be impacted by it. In the later phases of the project, there was a greater focus on stakeholders who have the greatest influence on the uptake of the training such as research funders and regulators.

**Fig. 1:**
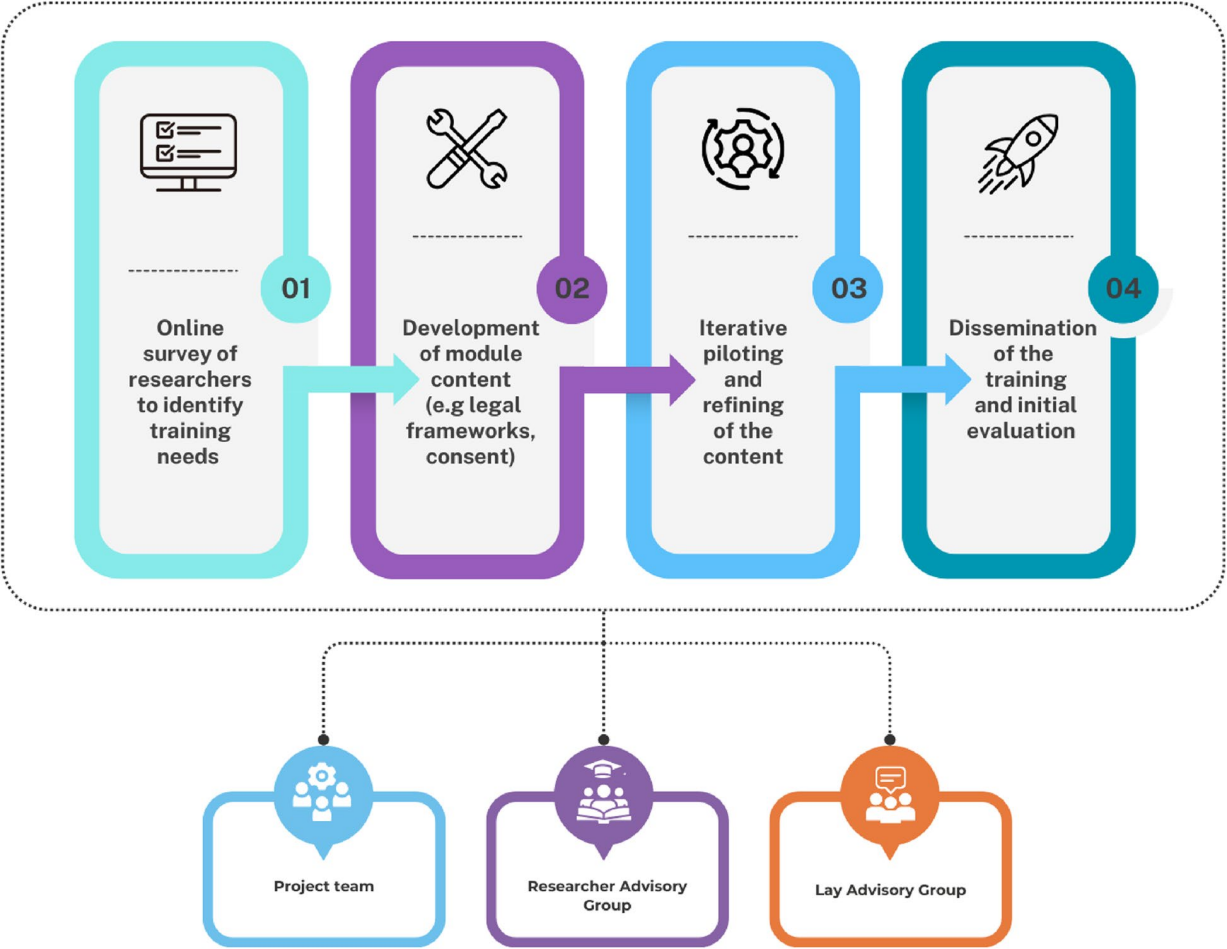
CONSULT Training development process

The project consisted of four phases: (1) a stakeholder survey to identify researchers’ training needs; (2) development of the CONSULT Training content; (3) iterative piloting and refining of the content; and (4) dissemination of the CONSULT Training and initial evaluation. The process is shown in Fig. 1 and the methods for each phase are described below. The findings from each phase are reported in the “Results” section that follows.

Ethical approval for the survey component of the project (phase 1) was provided by Cardiff University School of Medicine Research Ethics committee (Ref 23/80).

### Phase 1: stakeholder survey

In phase 1, a survey was conducted to explore researchers’ training and support needs, identify their learning preferences, and develop case studies and other curriculum content. The survey was aimed at UK researchers from a wide range of disciplinary backgrounds with experience of, or an interest in, research involving populations with capacity-affecting conditions. The survey was open to any researcher who designs and conducts trials that may involve the recruitment of adults with impaired capacity to consent in the UK. The survey questions were developed by the project team and refined in conjunction with feedback from the Lay and Researcher Advisory Groups.

Potential participants were identified through social media platforms (X/Twitter), research networks (e.g. MRC-NIHR Trials Methodology Research Partnership, UK Trial Managers’ Network, South West Research Hub) and groups (e.g. Dementia Researcher, British Society of Gerontology (BSG) Care Homes Research Special Interest Group) whose members design and conduct research involving adults lacking capacity. Networks and groups were asked to disseminate information about the study to their members via email or newsletter, including contact details for obtaining further information about the project. Databases of current and previously funded studies (e.g. NIHR portfolio) were also searched to identify researchers who are working in relevant areas.

The information shared via email or newsletter also contained a link to access the survey which was hosted on the Online Surveys platform (https://www.onlinesurveys.ac.uk/). The survey homepage contained participant information about the study, with a checkbox to confirm that respondents agreed to participate in the survey (a required field), followed by the questionnaire pages. See Appendix 1 for questionnaire pages. The questions were divided into four sections. The first section covered basic demographic data, followed by questions seeking free text information about the participant’s previous experience of training on research involving adults lacking capacity, their views about the current gaps in training and support on trials involving adults with impaired capacity to consent, the main challenges they encountered, and what they had found worked well. They were also asked about their experiences of public involvement in trials involving adults with impaired capacity across the lifecycle of a trial and provided with a list including whether the involvement was during the development of a funding application right through to disseminating the findings, with ‘yes’, ‘no’, and ‘not applicable’ response options for each item.

Descriptive statistics were used to summarise the quantitative survey responses. Participant responses in the free text boxes were analysed thematically using a coding frame developed by the research team [22], supported by the use of a qualitative analysis software tool (NVivo version 12) to help manage data.

### Phase 2: developing the e-learning content

The survey findings were then used to shape the e-learning content which was based on the CONSULT research programme led by the first author (VS) [18]. This included incorporating the findings from two previous studies: one identifying the barriers to conducting research involving adults with impaired capacity to consent [15] and a second that developed the INCLUDE Impaired Capacity to Consent Framework to help researchers to design trials including this population [19].

The content was also mapped to a wide range of resources that have been previously developed by the researchers involved in this project and other individuals/organisations (e.g. Health Research Authority). Collating resources as part of the training enabled the e-learning to also act as a desktop resource for researchers and other groups (e.g. Research Ethics Committee members) rather than being a one-off event to be completed.

The e-learning modules were built by the project team using a learning management system (LMS) which was selected following a review of candidate systems, with the Moodle Cloud platform (https://moodlecloud.com/) chosen to host the CONSULT Training based on functionality, acceptability, and cost. Users are required to register for a free account and are then able to access the self-guided training. This has enabled data to be collected on the number of users registering and on the completion rates of each module which helps to assess the reach and uptake of the training offer. This is also supported by other features such as users having the ability to download a certificate of completion, and completing an (optional) feedback survey that was built into the LMS to support initial evaluation of the e-learning.

### Phase 3: iterative piloting and refining the e-learning content

The content was iteratively piloted by members of the project team and Researcher Advisory Group who were representative of end users to establish its acceptability (e.g. order and flow of information, length of module content, ability to access without firewall restrictions), usability (e.g. embedded links, quizzes, playback of videos), and accessibility (e.g. font size, colour contrast). The content and design were then refined as required.

### Phase 4: dissemination and initial evaluation of the e-learning

The final phase of the project, to disseminate the e-learning and conduct an initial evaluation, was informed by a stakeholder analysis that was conducted during the initial set up of the project using the 3i framework [21]. This helped to identify the ‘parties’ (organisations and individuals) who are likely to have the greatest interest, influence, and impact, and so were important contacts to share information with about the e-learning and dissemination activities. Dissemination activities included a webinar that was planned to coincide with the launch of the e-learning, and the ability to pre-register for receiving the registration link for the e-learning once it was ‘live’.

## Results

### Phase 1: findings from the stakeholder survey

**Fig. 2:**
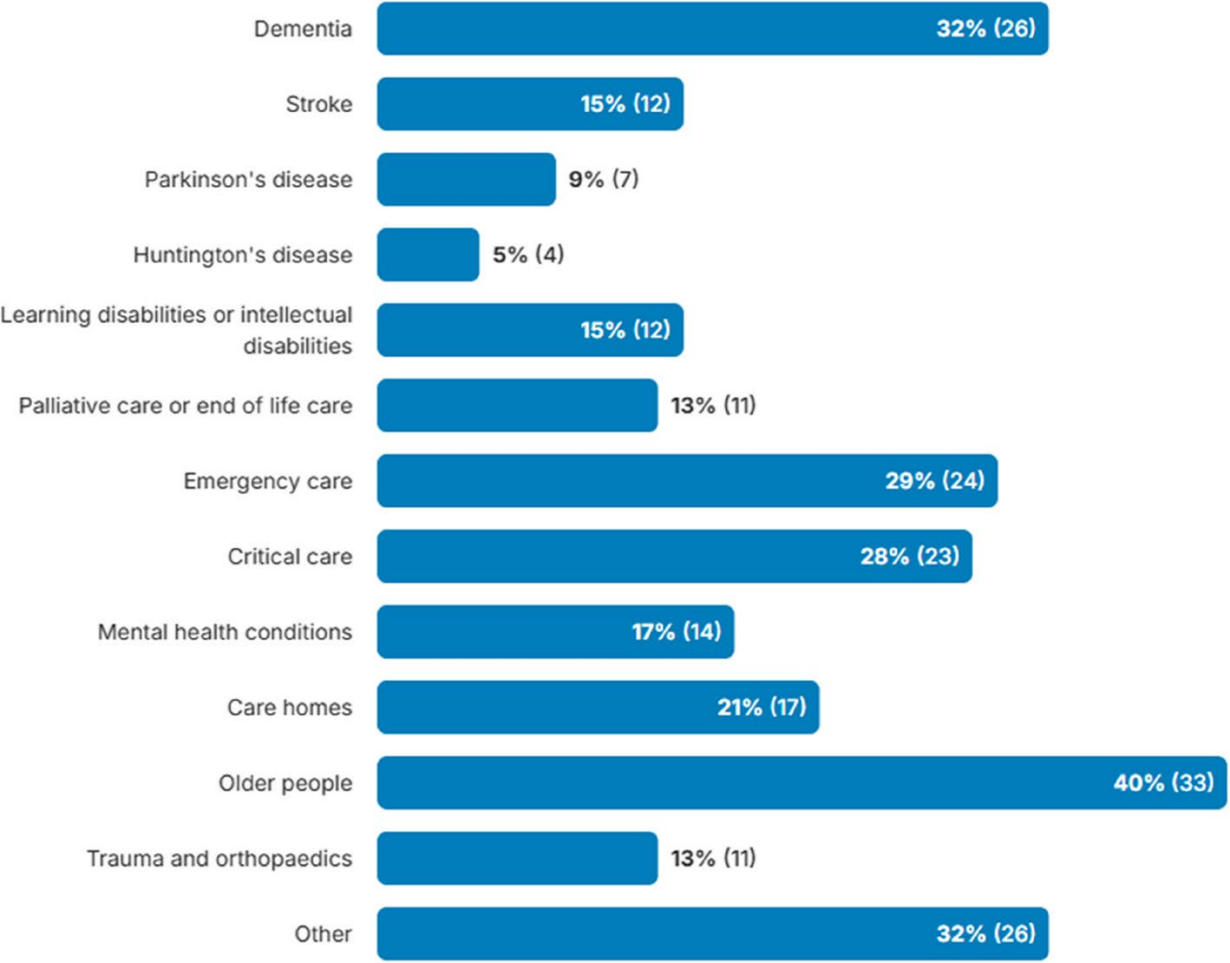
Research population or setting that the participant’s work concerns

The survey was open between November 2023 and January 2024. It was completed by 82 researchers or research teams from across the UK, the majority of whom were based in England (66%) and primarily worked in clinical trials units (CTUs) (33%) (see Table 1). Respondents had a range of roles in research including clinicians, CTU directors and trial/project managers, and their work spanned a broad range of populations (see Fig. 2).

**Table 1:** Stakeholder survey participant characteristics

Characteristic	No. of participants % (*n* = 82)
**Location**	
England	66% (54)
Northern Ireland	2% (2)
Scotland	15% (12)
Wales	16% (13)
Other	1% (1)
**Main employing organisation**	
UKCRC CTU*	33% (27)
NHS	26% (21)
Industry	1% (1)
Higher education institute/academic institution (non-CTU)	40% (33)
**Length of involvement with research (years)**	
0–5 years	15% (12)
6–10 years	26% (21)
11 + years	58% (48)
Unknown	1% (1)
**Activities included in participant’s role^**	
Designing trials	70% (57)
Conduct/management of trials	80% (66)
Approaching participants to take part in trials	45% (37)
Chief/principal investigator	38% (31)
Other	15% (12)
**Elements of a trial most involved in^**	
Trial management	68% (56)
Recruitment	61% (50)
Data management	44% (36)
Ethics	48% (39)
Statistics	12% (10)
Health economics	1% (1)
Other	20% (16)
**Current/previous projects involved adults with impaired capacity to consent**	
Yes	83% (68)
No	16% (13)
Other	1% (1)

^*^UK CRC CTU UK Clinical Research Collaboration registered Clinical trials Unit

^Participants could select all that applied

**Fig. 3:**
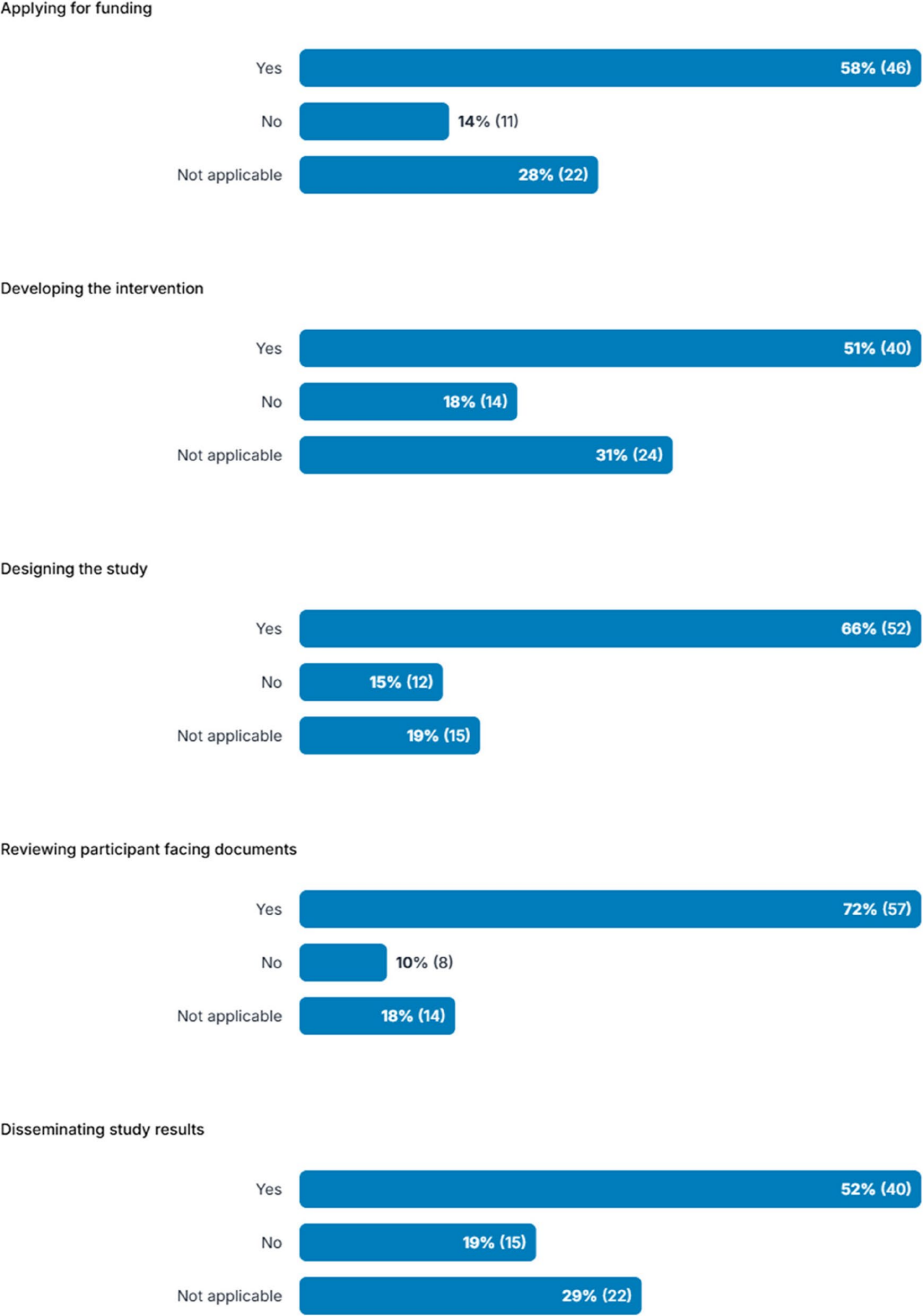
Participants’ experiences of involving members of the public, patients, or carers in different activities as part of any current or previous projects involving adults with impaired capacity

Participants were also asked about their experiences of involving public or patient contributors, and/or carers in any current or previous projects involving adults with impaired capacity to consent (see Fig. 3). Most had involved these groups when developing participant facing documents (72%), designing the study (65%), applying for funding (58%), developing the intervention (51%), and disseminating results (51%). Some participants had not involved public/patients/carers, ranging from 10 to 19% depending on the activity, and others indicated that it was ‘not applicable’ (18–31%) although not all participants had experience of studies involving adults with impaired capacity to consent. Free text responses explaining why they stated ‘no’ or ‘not applicable’ (which included both those who said they had experience of studies involving adults with impaired capacity to consent and those who did not) included that it was not part of the current stage of the project, their role only involved limited activities, or it had occurred prior to their involvement in the project.

#### Researchers’ training and support needs for designing and conducting trials involving adults with impaired capacity to consent

Participants were asked what previous training they had completed that was related to research involving people with impaired capacity to consent. This was a free text question and the most common response from the 79 participants who completed it was ‘none’ or ‘none at all’ (40%, *n* = 31 when the responses are combined). Others described accessing general Good Clinical Practice (GCP) training (20%, *n* = 16) or specific online training modules (14%, *n* = 11) such as the NIHR Informed Consent involving Adults Lacking Capacity course (both available via https://learn.nihr.ac.uk/), which were described by some as being ‘OK’, or providing ‘very broad overviews with little practical guidance’. Others reported undergoing study-specific training or completing training in specific aspects such as assessment of mental capacity as part of their clinical role.

Participants were asked if there were any gaps in training and support or if there were additional areas of training that they would find beneficial. Open text responses included updates on current legislation, providing accessible information and managing changes in capacity during a trial, information on undertaking capacity assessments, and ethical review processes.

When asked about their learning preferences from the list of options, participants considered some formats to be extremely useful, including top tips (76%), case studies (72%), watching videos (56%), additional resources such as websites (55%), and forums/discussion groups (48%), with other formats such as blogs and audio clips viewed less favourably.

#### Researchers’ experience of designing and conducting trials involving adults with impaired capacity to consent

**Fig. 4:**
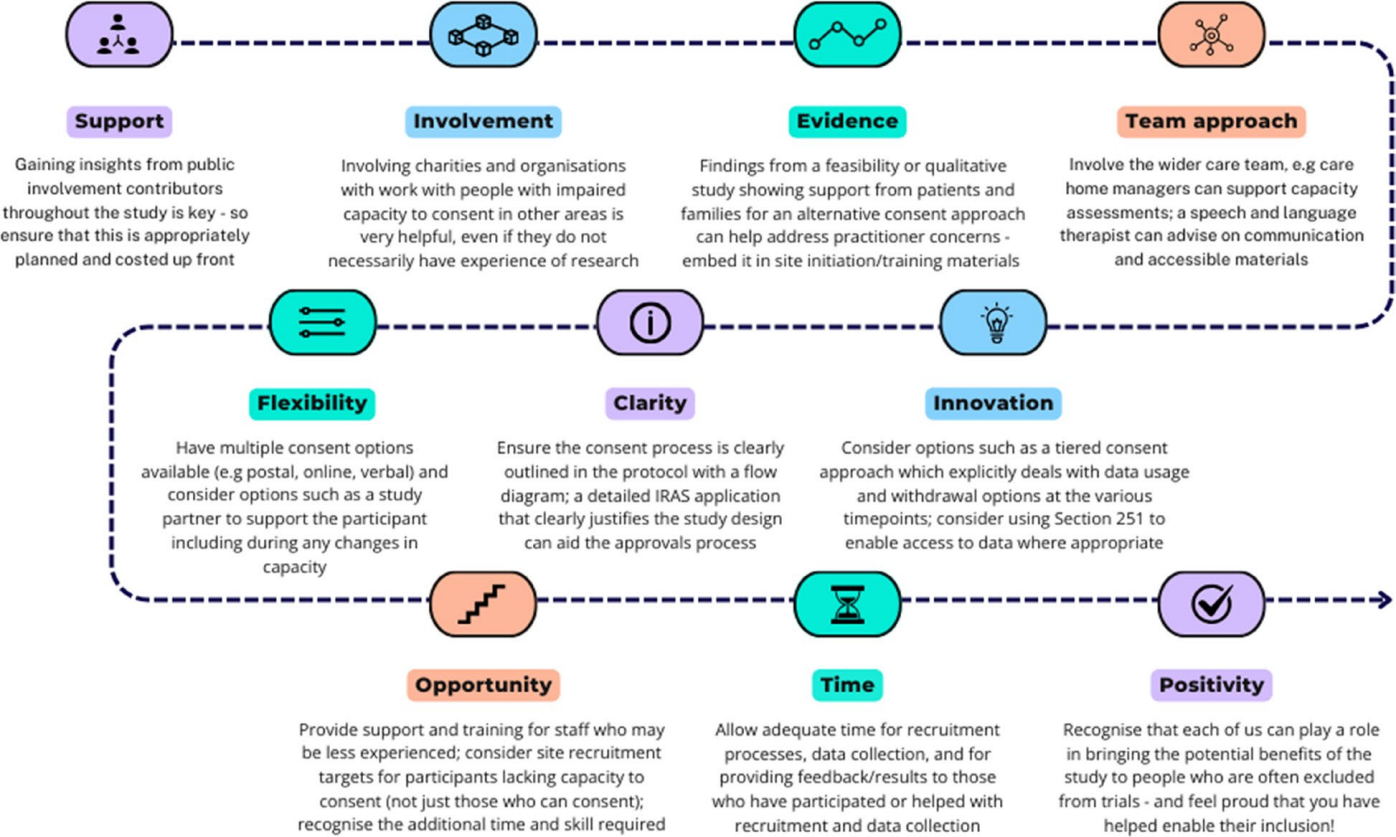
Summary of practical strategies that worked well when planning, setting up, and recruiting to a trial involving adults with impaired capacity to consent

Participants described a range of challenges they encountered when designing and conducting trials involving adults with impaired capacity to consent, including the differences between the legal frameworks governing different populations and parts of the UK, the corresponding differences in governance arrangements including ethical approvals. They described a range of strategies that they had used to try and address them, and their experience of public involvement in these trials and the valuable role this played. A thematic summary of the challenges encountered is provided in Table 2 which has been mapped to the phases of trial design and conduct identified in a previous study [15], and the practical strategies that the participants said they found worked well are summarised in Fig. 4.

**Table 2:** Summary of the key challenges encountered by participants when planning, setting up, and recruiting to a trial involving adults with impaired capacity to consent

Making trial design decisions
• Designing consent models and pathways for adults lacking capacity to consent • Intervention design, and establishing acceptability and feasibility for this population • Concerns about time costs and resources involved in recruiting this population • Appropriate outcome measures and data collection methods for this population • Sample size considerations, including participation changes due to loss of capacity during a trial
**Navigating ethical approval**
• Complexity of the legal frameworks including differing regulations/jurisdictions • Difficulties with data access and governance issues specific to this population • Justification for including adults lacking capacity and the lack of available data to support • Perceptions of risk for studies involving this population (including any risks to participants and the risk of needing additional time/resource for an application involving adults lacking capacity compared with a study that does not)
**Informing and supporting the participant**
• Lack of awareness about research and gatekeeping practices by carers and others • Skills and resources needed for creating accessible information
**Assessment of capacity to consent**
• Lack of tools and guidance for assessing capacity to consent • Skills and experience needed by those who are responsible for assessing capacity
**Involving an alternative decision-maker**
• Processes around the involvement of consultees and legal representatives • Additional documents required for alternative consent processes
**Revisiting consent and consultation**
• Loss of capacity during a trial and the processes required to enable continued participation

### Phase 2: developing the stakeholder-informed e-learning content

**Fig. 5:**
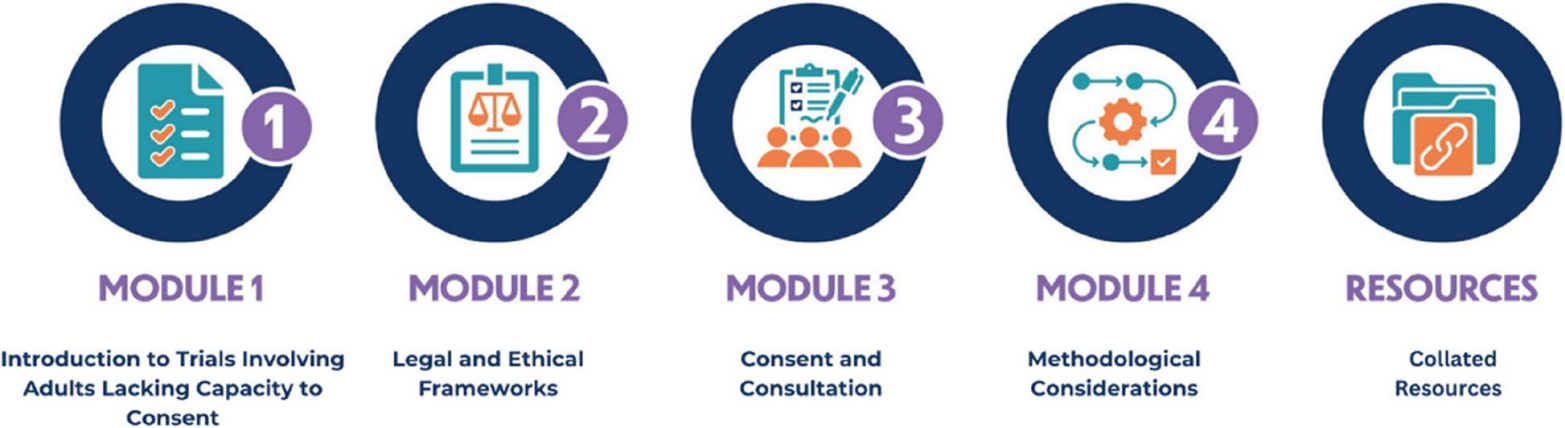
CONSULT Training e-learning modules

Informed by the survey findings and the wider CONSULT research programme [18], the e-learning content was developed across four modules (as shown in Fig. 5) with an additional section that contained key resources which were collated from across the modules.

**Fig. 6:**
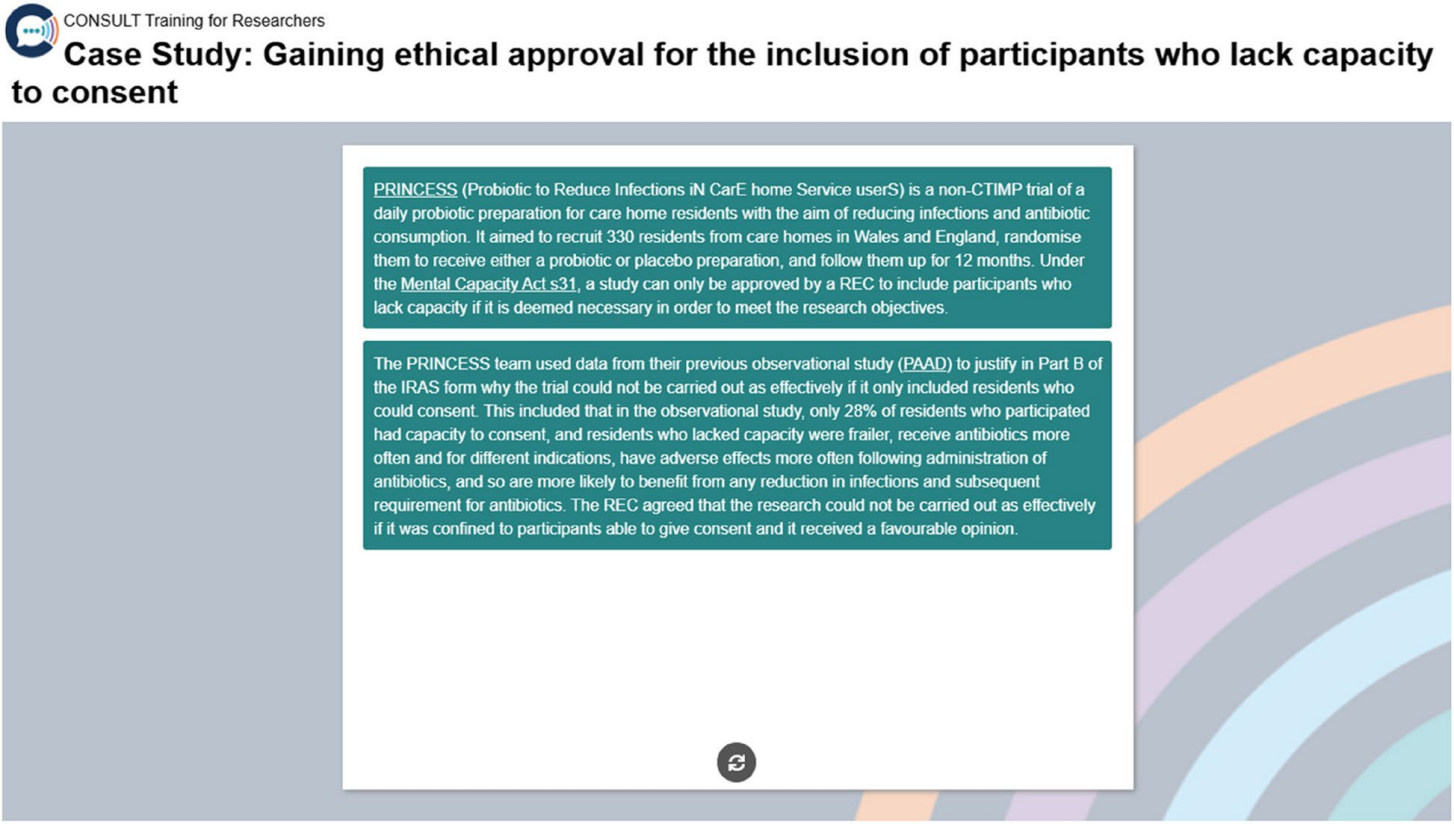
A case study used as part of CONSULT Training

Case studies, brief videos, and examples of best practice were developed to help illustrate key content based on survey responses alongside the experiences of the members of the project team and Researcher Advisory Group who conduct clinical trials involving this population. Examples include how a clinical trial justified the inclusion of adults lacking capacity during the ethical approvals process (see Fig. 6), and how evidence-based tools and accessible information have supported people with impaired communication and/or capacity to be involved in making decisions about research participation.

The lay advisory group were involved throughout to ensure that public involvement perspectives were threaded throughout the content, highlighting the importance of a person-centred approach to research involving this population. This included developing case studies highlighting the role of public involvement when seeking ethical approval for a trial involving adults lacking capacity and a video where one of the members of the lay advisory group shares their own personal experiences and explains why involving people with lived experience is particularly important when designing and conducting trials involving this population.

### Phase 3: refining the e-learning content based on feedback

The content was added to the LMS platform (Moodle) and iteratively tested by the project team, who refined the format of the content and the functionality of each module in order to improve the user experience. Prior to being finalised, the e-learning was piloted in its entirety by another member of the project team who had not previously used the LMS platform. Their feedback showed that the registration process had been straightforward; the modules had been easy to navigate through and enabled us to identify where any URL links embedded in the e-learning content were not working correctly.

Piloting the e-learning also provided us with the approximate time it had taken to complete each module, which ranged from under 10 min for the brief ‘Welcome’ section, through to 60–70 min to complete module 2, which is the longest of all the modules. Together, this means that it takes an estimated half a day to complete the CONSULT Training as a whole, including accessing the links to more information where appropriate.

This information also helped us to develop a series of infographics and guidance documents to help raise awareness about the CONSULT Training and to help users to register for and access the e-learning modules, with the aim of supporting dissemination and uptake.

### Phase 4: launch of the CONSULT Training and initial evaluation

The CONSULT Training e-learning was launched in December 2024 and is available online: www.capacityconsentresearch.com/training. The e-learning is designed to help researchers to understand how the legal and ethical frameworks governing trials involving adults lacking capacity apply in practice and learn more about the methodological and practical considerations for trials involving this population. It is intended for those who have experience with these trials, as well as those who are interested in learning more about the topic. The course is intended to complement other training that is available for researchers (e.g. NIHR Informed Consent With Adults Lacking Capacity) and resources that have previously been developed for researchers, including the CONSULT website of collated resources on capacity and consent and the NIHR INCLUDE Impaired Capacity to Consent Framework—a tool to help researchers to design studies that include people with impaired capacity to consent [19]. These are signposted throughout the modules and in the collated resources section. The CONSULT Training modules can be accessed at any time and are self-paced. Short quiz questions are provided at the end of each module to support self-assessment of the learning outcomes and consolidate learning. A certificate can be downloaded as a record of completing the training.

To coincide with the launch, we held a webinar titled ‘Addressing the Knowledge Gap’. Details about the webinar were shared through relevant organisations (e.g. UKCRC CTU Network, UK Trial Managers’ Network, BSG) and via social media (Twitter/X) informed by the stakeholder analysis conducted during the setup stage of the project, with 160 registered attendees including researchers, members of the public, and research ethics committee members.

The agenda was informed by the stakeholder survey which showed that researchers would value opportunities to discuss the issues they encounter, and by similar findings in the feedback from our previous webinar to launch the INCLUDE Impaired Capacity to Consent Framework. To provide background context, there were short presentations from the Director of the UK CRC CTU Network and members of the project team and a member of the lay advisory group shared her personal reflections. An expert panel then responded to questions raised by webinar attendees (some were submitted ahead of the webinar) who shared some of the key challenges they encountered. The webinar was recorded for those wishing to watch it back.

Following the webinar, an evaluation survey was distributed by email to attendees to explore their feedback about the webinar and their initial views about the e-learning. A short survey was created using Microsoft Forms (https://support.microsoft.com/en-us/forms) and consisted of a combination of multiple-choice options and open text responses (see Appendix 2). Characteristics of attendees who responded to the survey (*n* = 29) and their main area of interest are shown in Table 3. The survey questions are shown in Table 4 together with a thematic summary of the responses. Attendees who responded to the survey reported that they had heard about the webinar via email (48%, *n* = 14), from a colleague (28%, *n* = 8), or via social media (28%, *n* = 8). They rated the webinar as either very good (76%, *n* = 22) or good (24%, *n* = 7) which was reflected in their open text responses to questions asking about the impact of the webinar. There was also support for further similar webinars to help translate other new methodological research into practice.

**Table 3:** Characteristics of evaluation survey participants

Characteristic	No. of participants % (*n* = 29)
**Location**	
England	55% (16)
Wales	34% (10)
Scotland	10% (3)
**Role**	
Researcher	62% (18)
Health or social care professional	24% (7)
Methodologist	17% (5)
Research Ethics Committee member	10% (3)
Member of public/patient/carer	7% (2)
Other	7% (2)
**Area of interest in this topic^**	
Dementia or other neurodegenerative condition	52% (15)
Mental health conditions	45% (13)
Learning disabilities	41% (12)
Stroke	21% (6)
Palliative or end of life care	28% (8)
Emergency or critical care	28% (8)
Other	14% (4)
**Prior involvement in trials^**	
Contributed to the delivery of a trial	76% (22)
Contributed to the design of a trial	55% (16)
Contributed to the sharing of trial findings	45% (13)
Been a participant	10% (3)
Other	10% (3)

^Participants could select all that applied

**Table 4:** Summary of responses to evaluation survey

What were the highlights/key learn points?
**• Inclusivity is a priority area** – the session had highlighted for some attendees that research inclusivity is now considered a key priority area for policymakers, including being a condition of funding for some research funders (e.g. NIHR) **• Structured support is available** – the increased awareness and access to training and support led to attendees feeling better equipped to design trials including adults with impaired capacity to consent and less ‘daunted’ **• Sharing practical insights** – the combination of presentations, signposting to resources, and panel Q&A that explored some of the challenges that are encountered in practice (and ways to address them) was thought to be particularly informative **• This is why we do research! –** attendees particularly valued hearing from a member of the lay advisory group, highlighting the importance of hearing diverse perspectives
**What are you planning to implement that is different?**
**• Committing to undertaking the training** – many attendees reported that they planned to complete the training and had already distributed the training to other members of their team and encouraged them to undertake it too **• Sharing the message more widely** – many attendees reported either having shared the information and resources with collaborators and other connections, or pledged action to do so **• Building evidence to justify inclusion** – beyond improvements in knowledge and understanding, attendees felt they would have a more justifiable argument to include adults with impaired capacity in future trials and better able to demonstrate why this is important for the validity of the results **• Influencing change at an organisational level** – attendees reported that they were exploring potentially implementing the training at an institutional level, or across several research ethics committees, and exploring the need for an inclusion role within their department

Following the launch, CONSULT e-learning has been accessed by 80 + registered users to date. Those who access the e-learning are invited to complete a short evaluation survey (see Appendix 3) which is built into the LMS to refine the module content and/or layout as required. Scores from those completing the survey to date (*n* = 6) indicate an increase in their perceived knowledge about how to design and conduct trials involving adults lacking capacity to consent before and after completing the CONSULT e-learning out of 10, with a change in mean score from 7.2 to 8.8. Analysis of the free text comments indicates that the e-learning is ‘an excellent resource’ that ‘complements our current informed consent training’ and ‘definitely fills a training need gap’. Key take-away messages included the legislative differences across the UK and the need to adapt trial design to support inclusion of adults with incapacity.

## Discussion

The CONSULT e-learning was stakeholder-informed and developed over four phases of the project in collaboration with a group of researchers with topic expertise and a lay advisory group with lived experience. It consists of a series of free-to-access modules which contain multi-media content, quiz questions to consolidate learning, case studies to illustrate key points, and links to more in depth information and practical resources if desired. As national and international legal and ethical frameworks are updated over time, and with increasing use of innovative trial designs, the contents may need to be periodically updated.

There are a number of strengths and limitations to this project. Whilst responses to the stakeholder survey were provided by researchers and research teams with a broad range of experiences and roles and working with a diverse range of populations and settings, the number of responses was relatively modest and are likely to represent individuals and teams who have an interest in inclusion of under-served groups. The post-webinar survey indicated an increased awareness amongst attendees and a commitment to changing practice; however, the proportion of attendees who completed the survey was relatively low, although comparable with previous events run by the research centre, and may be subject to response bias. The e-learning enables registered users to have unlimited access to modules and encourages them to access content flexibly and to use it as a desktop resource; therefore, it is not possible to accurately report completion rates. However, approx. 19 of the 80 + registered users have marked all sections as complete and downloaded a certificate of completion.

Following the successful launch of the CONSULT e-learning, there is now a focus on wider dissemination through engaging with key organisations such as research funders and the UK Clinical Research Collaboration (UKCRC) Registered Clinical Trials Unit (CTU) Network which has a strategic priority to improve diversity and inclusivity in trials and to train and support CTU members [23]. In parallel work (OPTIMISE), we are also developing a series of recommendations for researchers to help them to design more inclusive consent processes for people with additional communication and/or decision-making needs [24]. This will include mapping resources such as the CONSULT e-learning onto the recommendations to encourage uptake.

However, it is widely acknowledged that awareness alone does not necessarily initiate or sustain changes in behaviour or practice [25]. Ultimately, we need to tackle the system-wide barriers to change in order to address the wider ethical, legal, and methodological challenges that limit the inclusion of adults with impaired capacity to consent [15]. Future work will include more formally evaluating the implementation and effectiveness of the CONSULT e-learning, alongside a larger programme of work (ACCORD) to develop behaviourally informed interventions to support more inclusive trials involving this population [26].

## Conclusions

In response to the growing focus on improving research inclusion, and the calls for better training and awareness about how to improve the inclusion of under-served groups in research, we developed the CONSULT e-learning to support researchers and other groups to design and conduct trials involving adults with impaired capacity to capacity to consent. Alongside tools such as the INCLUDE Impaired Capacity to Consent Framework, the CONSULT e-learning course aims to enable researchers to develop the knowledge and skills needed to design and conduct higher-quality trials that are more inclusive of adults with impaired capacity to consent. Key messages include the need for evidence-based, flexible, and innovative approaches to clinical trial design, supported by diverse public involvement.

Initial evaluation has indicated that the training is welcomed by researchers, health and social care professionals, and research ethics committee members, who have pledged to change their practice as a result. Further engagement, including with funders who are increasingly requiring inclusion as a condition of funding, is needed. The contents may require updating to reflect incoming regulatory changes to UK clinical trials, and the e-learning content may prove to be adaptable for other international clinical trial contexts.

## Supplementary Information

Supplementary Material 1.

Supplementary Material 2.

Supplementary Material 3.

## Data Availability

The CONSULT e-learning is available online at www.capacityconsentresearch.com/training.

## Abbreviations

BSG: British Society of Gerontology
HRA: Health Research Authority
NIHR: National Institute for Health and Care Research
RCT: Randomised controlled trial
REC: Research ethics committee
UKCRC CTU: UK Clinical Research Collaboration Clinical Trials Unit
UKTMN: UK Trial Management Network
