# Supplementary material for: Addressing the knowledge gap: development of stakeholder-informed training to improve the inclusion of adults with impaired capacity to consent in trials
Source: Trials. 2025 Oct 22;26:429. doi: 10.1186/s13063-025-09182-1 (PMC12542349; doi:10.1186/s13063-025-09182-1)
Supplement: Supplementary file 1 — Supplementary Material 1. [file 13063_2025_9182_MOESM1_ESM.docx]

**Development of stakeholder-informed training for researchers designing and conducting trials involving adults with impaired capacity to consent (CONSULT Training)**

# **Part 1. About you**

1. **Which group best describes your current main employing organisation?**

- UK CRC registered Clinical Trials Unit (CTU)
- NHS
- Social care
- Industry
- HEI/academic institution (non-CTU)
- Other (*please specify*)

1. **What is your current role/job title?**
2. **Where do you work?**

- England
- Northern Ireland
- Scotland
- Wales
- Other (*please specify*)

1. **How long have you been involved in research?**

- 0-5 years
- 6-10 years
- 11+ years
- Other (*please specify*)

1. **Which setting or population does your work concern? (Please select all that apply)**

- Dementia
- Stroke
- Parkinsons disease
- Huntington’s disease
- Learning disabilities or intellectual disabilities
- Palliative care or end of life care
- Emergency care
- Critical care
- Mental health conditions
- Care homes
- Older people
- Trauma and orthopaedics
- Other (*please specify*)

1. **Does your role include designing trials?**

- Yes
- No
- Other (*please specifiy*)

1. **Does your role include the conduct/management of trials?**

- Yes
- No
- Other (*please specifiy*)

1. **Does your role include approaching participants to take part in trials?**

- Yes
- No
- Other (*please specifiy*)

1. **Which of these elements of a trial are you most involved in? (Please select all that apply)**

- Health economics
- Ethics
- Statisitics
- Trial management
- Recruitment
- Data managment
- Other (*please specifiy*)

1. **Have you worked on any current or previous projects involving adults with impaired capacity to consent?**

- Yes
- No
- Other (*please specifiy*)

# **Part 2. Researcher training needs assessment – experiences of training**

**In this section, we would like to know about the training you have received to help you with designing and/or conducting research involving people with impaired capacity to consent.**

1. What formal training have you taken part in related to research involving people with impaired capacity to consent? If possible please provide the training provider, a brief overview of the content and your appraisal of the training. (i.e any training provided externally or inhouse, including online training)
2. What other forms of support have you received related to research involving people with impaired capacity to consent? (e.g. networks, workshops, seminars)
3. Are there any important gaps in training/support currently available, or additional areas of training that you would find beneficial?

# **Part 3. Researcher training needs assessment – experiences of studies**

**In this section, we would like to know about any challenges and positive experiences you may have encountered when designing and/or conducting trials involving people with impaired capacity to consent.**

1. What key challenges do you face in planning a trial involving people with impaired capacity to consent? (i.e. when designing the study at grant application stage and/or writing the protocol)?
2. What key challenges do you face when setting up a trial involving people with impaired capacity to consent? (i.e. when liaising with sites and external organisations, gaining regulatory approvals)?
3. What key challenges do you face in recruiting participants to a trial involving people with impaired capacity to consent? (i.e. when assessing eligibility, identifying participants, alternative consent processes)?
4. What key challenges do you face in retaining participants in a trial involving people with impaired capacity to consent? (i.e. when capacity status changes during participation)?
5. What key challenges do you face in collecting data in a trial involving people with impaired capacity to consent? (i.e. outcome measurement instruments, format, completion)?
6. What key challenges do you face in analysing, reporting and disseminating a trial involving people with impaired capacity to consent? (i.e. sub-group analysis, sharing the results with participants and others)?
7. Are there other aspects of designing and/or conducting a trial involving people with impaired capacity to consent that hasn’t already been covered above that you have had challenges with?
8. We are also very interested in what goes well when designing and conducting a trial involving people with impaired capacity to consent. Please tell us about positive examples, what made them positive and any recommendations for other researchers. This could be at any stage of the project such as planning, recruitment, dissemination etc.

# **Part 4. Researcher training needs assessment – experiences of public involvement**

**We are interested in your experiences of working with members of the public, patients and carers when designing and conducting studies involving people with impaired capacity to consent. By** [**public involvement**](https://www.nihr.ac.uk/documents/briefing-notes-for-researchers-public-involvement-in-nhs-health-and-social-care-research/27371) **we mean actively involving them in the research process itself rather than being participants in research.**

1. Have you involved public/patients/carers in the following activities in any of your current or previous projects involving adults with impaired capacity to consent? (Please select ONE box every line, if N/A please explain why in text box provided below)

|  | **Yes** | **No** | **Not applicable** |
| --- | --- | --- | --- |
| Applying for funding | ꙱ | ꙱ | ꙱ |
| Developing the intervention | ꙱ | ꙱ | ꙱ |
| Designing the trial | ꙱ | ꙱ | ꙱ |
| Reviewing participant facing documents | ꙱ | ꙱ | ꙱ |
| Disseminating study results | ꙱ | ꙱ | ꙱ |
| Please explain why in the text box provided if you have selected not applicable for any of the above activities)? | | | |

1. When you have undertaken public involvement activities in trials involving adults with impaired capacity to consent, were there aspects that did not work well? Please consider all stages of the research project (planning, applying, disseminating etc) and consider issues around accessibility.
2. What did work well? Please consider all stages of the research project (planning, applying, disseminating etc) and consider issues around accessibility.

# **Part 5. Researcher training needs assessment – learning preferences**

**Finally, we would like to know how you prefer to learn - this will help guide us when designing the training programme.**

How useful do you find (or think you would find) the following methods of learning when completing an online learning course? (Please select ONE box on EVERY line)

|  | **Extremely useful** | **Somewhat useful** | **Not at all useful** |
| --- | --- | --- | --- |
| Forums/Discussion groups | ꙱ | ꙱ | ꙱ |
| Evaluation questions/Quiz | ꙱ | ꙱ | ꙱ |
| Top tips | ꙱ | ꙱ | ꙱ |
| Watching videos | ꙱ | ꙱ | ꙱ |
| Reading snippets of text/articles | ꙱ | ꙱ | ꙱ |
| Reading blogs | ꙱ | ꙱ | ꙱ |
| Listening to audio clips | ꙱ | ꙱ | ꙱ |
| Assignments (online tasks/activites) | ꙱ | ꙱ | ꙱ |
| Case studies | ꙱ | ꙱ | ꙱ |
| Additional resources (website and lesson files) | ꙱ | ꙱ | ꙱ |
| Are there any other online training methods you have found useful (please specify)? | | | |
