# Supplementary material for: Addressing the knowledge gap: development of stakeholder-informed training to improve the inclusion of adults with impaired capacity to consent in trials
Source: Trials. 2025 Oct 22;26:429. doi: 10.1186/s13063-025-09182-1 (PMC12542349; doi:10.1186/s13063-025-09182-1)
Supplement: Supplementary file 2 — Supplementary Material 2. [file 13063_2025_9182_MOESM2_ESM.docx]

**CONSULT Training for Researchers Webinar – feedback survey questions**

**Q1. Did you attend the webinar as?**

- Health or social care professional
- Researcher
- Methodologist
- Policy maker
- Member of the public/patient/carer
- Research ethics committee member
- Other (please specify)

**Q2. Which best describes your area of interest in this topic? Tick all that apply**

- Stroke
- Dementia or other neurodegenerative conditions
- Learning disabilities
- Mental health conditions
- Palliative or end of life care
- Emergency or critical care
- Other (please specify)

**Q3. What prior involvement have you had in trials? Tick all that apply**

- Contributed to the delivery of a trial
- Contributed to sharing of trial findings
- Contributed to the design of a trial
- Been a participant
- Other (please specify)

**Q4. Where are you from?**

- Wales
- England
- Scotland
- Northern Ireland
- Other

**Q5. How did you hear about this webinar?**

- Newsletter
- Friend/Colleague
- Email
- Twitter X
- Other

**Q4. How would you rate your overall experience of the webinar?**

- Very good
- Good
- Satisfactory
- Poor
- Very poor

**Q5. Please tell us more about your experience of the webinar.**

**Q6. What were the highlights of the event for you. For example, anything you learned?**

**Q7. What are you planning to implement that’s different?**

**Q8.** **Do you have any other comments, or any feedback that would be helpful for future events**?
