# Supplementary material for: Addressing the knowledge gap: development of stakeholder-informed training to improve the inclusion of adults with impaired capacity to consent in trials
Source: Trials. 2025 Oct 22;26:429. doi: 10.1186/s13063-025-09182-1 (PMC12542349; doi:10.1186/s13063-025-09182-1)
Supplement: Supplementary file 3 — Supplementary Material 3. [file 13063_2025_9182_MOESM3_ESM.docx]

**CONSULT training - evaluation survey questions**

**Part 1. About you**

1. **Which group best describes your current main employing organisation?**

- UK CRC registered Clinical Trials Unit (CTU)
- NHS
- Social care
- Industry
- HEI/academic institution (non-CTU)
- Other (*please specify*)

1. **What is your current role/job title?**


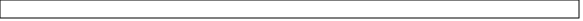


1. **Where do you work?**

- England
- Northern Ireland
- Scotland
- Wales
- Other (*please specify*)

1. **How long have you been involved in research?**

- 0-5 years
- 6-10 years
- 11+ years
- Other (*please specify*)

1. **Which setting or population does your work concern? (Please select all that apply)**

- Dementia
- Stroke
- Parkinsons disease
- Huntington’s disease
- Learning disabilities or intellectual disabilities
- Palliative care or end of life care
- Emergency care
- Critical care
- Mental health conditions
- Care homes
- Older people
- Trauma and orthopaedics
- Other (*please specify*)

1. **Does your role include designing trials?**

- Yes
- No
- Other (*please specify*)

1. **Does your role include the conduct/management of trials?**

- Yes
- No
- Other (*please specify*)

1. **Does your role include approaching participants to take part in trials?**

- Yes
- No
- Other (*please specify*)

1. **Which of these elements of a trial are you most involved in? (Please select all that apply)**

- Health economics
- Ethics
- Statistics
- Trial management
- Recruitment
- Data management
- Other (*please specify*)

1. **Have you worked on any current or previous projects involving adults with impaired capacity to consent?**

- Yes
- No
- Other (*please specify*)

1. **How did you hear about the CONSULT e-learning resource?**

- Twitter/X
- UK TMN
- UKCRC CTU network
- ICTMC
- Other (*please specify*)

**Part 2. Experiences of CONSULT training**

**How much of the CONSULT e-learning content did you access?**

- Welcome
- Module 1
- Module 2
- Module 3
- Module 4
- Other (*please specify*)

**Before completing the CONSULT e-learning, out of 10 how would you rate your knowledge about how to design and conduct trials involving adults lacking capacity to consent?**

<scale 1-10>

**After completing the CONSULT e-learning, out of 10 how would you rate your knowledge about how to design and conduct trials involving adults lacking capacity to consent?**

<scale 1-10>

**What are your main takeaways from the e-learning?**

<open text box>

**What are you planning to implement that’s different?**

<open text box>

**Any other feedback?**

<open text box>
